# Mutational landscape of gastric cancer and clinical application of genomic profiling based on target next-generation sequencing

**DOI:** 10.1186/s12967-019-1941-0

**Published:** 2019-06-04

**Authors:** Hui Cai, Changqing Jing, Xusheng Chang, Dan Ding, Ting Han, Junchi Yang, Zhengmao Lu, Xuguang Hu, Zhaorui Liu, Jinshen Wang, Liang Shang, Shouxin Wu, Peng Meng, Ling Lin, Jiangman Zhao, Mingming Nie, Kai Yin

**Affiliations:** 1 Department of Gastrointestinal Surgery, Changhai Hospital, The Second Military Medical University, 168 Changhai Road, Yangpu District, Shanghai, 200433 China; 2 Department of Gastrointestinal Surgery, Shandong Provincial Hospital Affiliated to Shandong University, Jinan, 250021 China; 3 Zhangjiang Center for Translational Medicine, Shanghai Biotecan Pharmaceuticals Co., Ltd., 180 Zhangheng Road, Shanghai, 201204 China; 4 Shanghai Zhangjiang Institute of Medical Innovation, Shanghai, 201204 China

**Keywords:** Gastric cancer, Next-generation sequencing, Tumor mutation burden, Clinical actionable alterations

## Abstract

**Background:**

Gastric cancer (GC) is a leading cause of cancer deaths, and an increased number of GC patients adopt to next-generation sequencing (NGS) to identify tumor genomic alterations for precision medicine.

**Methods:**

In this study, we established a hybridization capture-based NGS panel including 612 cancer-associated genes, and collected sequencing data of tumors and matched bloods from 153 gastric cancer patients. We performed comprehensive analysis of these sequencing and clinical data.

**Results:**

35 significantly mutated genes were identified such as *TP53*, *AKAP9*, *DRD2*, *PTEN*, *CDH1*, *LRP2* et al. Among them, 29 genes were novel significantly mutated genes compared with TCGA study. *TP53* is the top frequently mutated gene, and tends to mutate in male (p = 0.025) patients and patients whose tumor located in cardia (p = 0.011). High tumor mutation burden (TMB) gathered in *TP53* wild-type tumors (p = 0.045). TMB was also significantly associated with DNA damage repair (DDR) genes genotype (p = 0.047), Lauren classification (p = 1.5e−5), differentiation (1.9e−7), and HER2 status (p = 0.023). 38.31% of gastric cancer patients harbored at least one actionable alteration according to OncoKB database.

**Conclusions:**

We drew a comprehensive mutational landscape of 153 gastric tumors and demonstrated utility of target next-generation sequencing to guide clinical management.

**Electronic supplementary material:**

The online version of this article (10.1186/s12967-019-1941-0) contains supplementary material, which is available to authorized users.

## Background

Gastric cancer (GC) is the third leading cause of cancer-related mortality in the world, with the highest incidence occurring in Eastern Asia (particularly in China) and lowest in Northern America [1]. In China, there were 679,100 new cases and 498,000 deaths of gastric cancer in 2015, which account for above half of the world’s gastric cancer deaths [2]. Gastric cancer is a heterogeneous disease with phenotypic diversity that encompasses various molecular subtypes, which can be subdivided into intestinal and diffuse types according to the Lauren classification [3]. Recent studies using next-generation sequencing (NGS) have revealed an extensive repertoire of potential cancer-driving genes and drew mutational landscape of gastric cancer. The Cancer Genome Atlas (TCGA) project divided gastric cancer into four subtypes: Epstein–Barr virus (EBV) positive, microsatellite instability (MSI), genomically stable (GS) and chromosomal instability (CIN) [4]. Chen et al. performed whole-exome sequencing (WES) on paired normal-cancer tissues of 78 gastric cancer patients in northern China (Tianjin), which distinguished two GC subtypes with either high-clonality (HiC) or low-clonality (LoC) [5].

Over the last decade, NGS has enabled application of clinical genomics to the diagnosis and treatment of cancers. Taking lung cancer as an example, it has become standard practice to profile tumors for driver mutations. Target capture sequencing may identify actionable mutated driver genes and further determine tumor mutation burden (TMB), which is more cost effective and efficient, and achieves higher sequencing depth than WES. Zehir et al. have reported the mutational landscape of 10,000 pan-cancer patients using a hybridization capture-based NGS panel (MSK-IMPACT) [6]. In recent years, a number of studies about gastric cancer adopted target next-generation sequencing technology [7–9].

We developed a hybridization capture-based NGS panel, Biotecan PanCancer Panoramic Detection (BTC-PCPD), which is capable of detecting protein-coding mutations, copy number alterations (CNAs), TMB and selected promoter mutations in 612 cancer-associated genes (2.75 M) (Additional file 1). BTC-PCPD panel was designed referring to cancer-related database, clinical guidelines, and high-quality references, which included cancer genetic risk genes, targeted drugs (approved by FDA, clinical trials) and chemotherapy associated genes, and prognosis genes (Additional file 1). In this study, we performed BTC-PCPD panel sequencing on tumors and paired peripheral blood from 153 patients with gastric tumor. Using these data, we drew a comprehensive mutational landscape of 153 gastric tumors and demonstrated utility of massively parallel DNA sequencing of tumors to guide clinical management.

## Methods

### Patients and samples

We recruited 153 gastric cancer patients from Department of Gastrointestinal Surgery in Changhai Hospital (Shanghai, China) and Department of Gastrointestinal Surgery in Shandong Provincial Hospital (Jinan, China) between February 2017 and November 2018. 134 fresh frozen tumor tissues and 19 Formalin-Fixed and Paraffin-Embedded (FFPE) tumor tissues were collected after surgery. We used germline DNA from blood as a reference for detecting somatic alterations. Clinical characteristics of 153 gastric cancer patients were showed in Table 1.

**Table 1:** Clinical characteristics of patients enrolled in this study according to *TP53* status

Clinical characteristics	No. of patients	*TP53*		p value
		Wild type	Mutated	
Total	153	63 (41.18%)	90 (58.82%)	
Gender				
Male	103	36	67	0.025*
Female	50	27	23	
Age median (range)	59 (19–80)			
Stage				
I	25	12	13	0.206
II	39	18	21	
III	69	22	47	
Lauren classification				
Diffuse	50	23	27	0.163
Intestinal	47	13	34	
Mixed	24	10	14	
Differentiation				
Poorly	62	29	33	0.21
Moderately and poorly	27	9	18	
Moderately	45	14	31	
Location				
Cardia	24	4	20	0.011*
Gastric (fundus, body, antrum, pylorus)	122	56	66	
Lymphatic metastasis				
Yes	89	31	58	0.067
No	47	24	23	
Drinking				
Yes	21	6	15	0.228
No	127	54	73	
Smoke				
Yes	30	10	20	0.368
No	118	50	68	
HER-2 IHC				
Positive	11	2	9	0.208
Negative	110	43	67	

Patients were not showed whose clinical data was missing

* p < 0.05, ** p < 0.01, *** p < 0.001

### DNA extraction and quality control

gDNA from fresh tissue was extracted by QIAamp DNA Mini Kit, gDNA from blood by QIAamp DNA Blood Mini Kit, gDNA from FFPE tissues by GeneRead DNA FFPE Kit (Qiagen, Hilden, Germany). Quantity and purity of gDNA were assessed by Qubit^®^ 3.0 Fluorometer (Invitrogen, Carlsbad, CA, USA) and NanoDrop ND-1000 (Thermo Scientific, Wilmington, DE, USA). Fragmentation status were evaluated by the Agilent 2200 TapeStation system using the Genomic DNA ScreenTape assay (Agilent Technologies, Santa Clara, CA, USA) able to produce a DNA Integrity Number (DIN). An additional quality control (QC) step to assess FFPE DNA integrity was performed using a multiplex Polymerase Chain Reaction (PCR) approach [10]. Briefly, 30 ng of gDNA were amplified using three different-size set of primers of glyceraldehyde-3-phosphate dehydrogenase (GAPDH) gene (200–300–400 base pair), and the concentration of PCR products was determined by Agilent 2100 Bioanalyzer instrument (Agilent Technologies). Then, to estimate FFPE gDNA fragmentation, we evaluated an Average Yield Ratio (AYR) value, calculated by yield ratio of each amplicon compared with a reference DNA (Promega Madison, WI, USA).

### Library preparation and hybridization capture

A total of 300 ng of each gDNA sample based on Qubit quantification were mechanically fragmented on a E220 focused ultrasonicator Covaris (Covaris, Woburn, MA, USA). Two hundred nanogram of sheared gDNA were used to perform end repair, A-tailing and adapter ligation with either Agilent SureSelect XT (Agilent Technologies) or KAPA library preparation kits (KapaBiosystems Inc. Wilmington, MA, USA), following the manufacturer instructions. Subsequently, the libraries were captured using Agilent SureSelect XT custom 0.5–2.9 M (Agilent Technologies) probes, and finally amplified.

### Clustering and sequencing

After QC and quantification by Agilent 2100 Bioanalyzer (Agilent Technologies) and Qubit^®^ 2.0 Fluorometer (Invitrogen), the libraries were sequenced on an Illumina Next 500 platform (IlluminaInc, San Diego, CA, USA) High Output mode, 2 × 75 cycles.

### Bioinformatics analysis

Clean data was obtained following filtering adapter, low quality reads and reads with proportion of N > 10%. Reads were aligned to the reference human genome (UCSC hg19) using the Burrows–Wheeler Aligner v. 0.7.12. Next, the Picard and Genome Analysis Toolkit (GATK v.3.2) method was adopted for duplicate removal, local realignment and Base Quality Score Recalibration, and generated the quality statistics, including mapped reads, mean mapping quality and mean coverage. Finally, the GATK HaplotypeCaller was used for SNV and InDel identification.

Variants were annotated using the ANNOVAR software tool. Annotations for mutation function (including frameshift insertion/deletion, non-frameshift insertion/deletion, synonymous SNV, nonsynonymous SNV, stop gain stop loss), mutation location [such as exonic, intronic, splicing, upstream, downstream, 3′ untranslated region (UTR), 5′UTR and so on], amino acid changes, 1000 Genomes Project data and dbSNP reference number were performed.

Somatic SNVs and InDels of tumors compared with matched normal tissue were named and functionally annotated using MuTect v. 1.1.4 and Varscan2 v. 2.3.9 software. The mutations with variant allele frequency > 5% were defined as high confidence mutations. Tumor mutation burden (TMB) was defined as the number of all somatic base substitution and indel per mega base excluding synonymous mutation.

MutSigCV v.0.9 [11] was used to identify significantly mutated genes (q < 0.1). Then, gene mutation data were downloaded from TCGA database, and comparative analysis was performed using the sequencing data produced in the present study. Varscan2 software was used for identifying and annotating CNAs (|log2_ratio| > 1). To account for differences in sequence data input, the ratio of tumor depth to normal depth was normalized with the data-ratio parameter. The relative copy number in tumor in computed from the log-base-2 of the ratio of tumor depth to normal depth.

### Statistical analysis

The mutation landscape across a cohort, including SNVs, InDels and mutational burden, were created by Genomic Visualizations in R (GenVisR). The custom mutation lists of proteins were visualized by MutationMapper tool from cBioPortal. Gene ontology (GO) and Kyoto Encyclopedia of Genes and Genomes (KEGG) enrichment analysis were performed to investigate the biological importance of the somatic mutated genes of all samples using the ClusterProfiler in R software [12]. The cutoff of p-values < 0.05 and FDR < 0.05 were used to assess the significance of enrichment terms. The nonparametric Mann–Whitney U test was subsequently used to test for significance in difference of means between two populations.

## Results

### Characteristics of patients and sequencing data

Clinical characteristics of 153 gastric cancer patients were showed in Table 1. Primary tumors were from the following anatomic locations of the stomach: 24 from cardia, 64 from the antrum, 36 from the body, 4 from the pylorus, 3 from the fundus, and 15 across over two locations. All histopathologic diagnoses were subjected to independent reviews by at least two senior pathologists. According to Lauren classification, 50 cases were classified as diffuse-type, 47 as intestinal-type, and 24 as mixed-type. Among these patients, 25 were stage I, 39 were stage II, and 69 were stage III.

Massively parallel DNA sequencing achieved an average of 594 × coverage of the tumor genomes with 0.91 of Q30, and 142 × coverage of the germline genomes with 0.91 of Q30. There was no significant difference of sequencing depth and Q30 between fresh tissues and FFPE tissues, which showed FFPE tissues obtained good sequence data.

### Landscape of somatic mutations

**Fig. 1:**
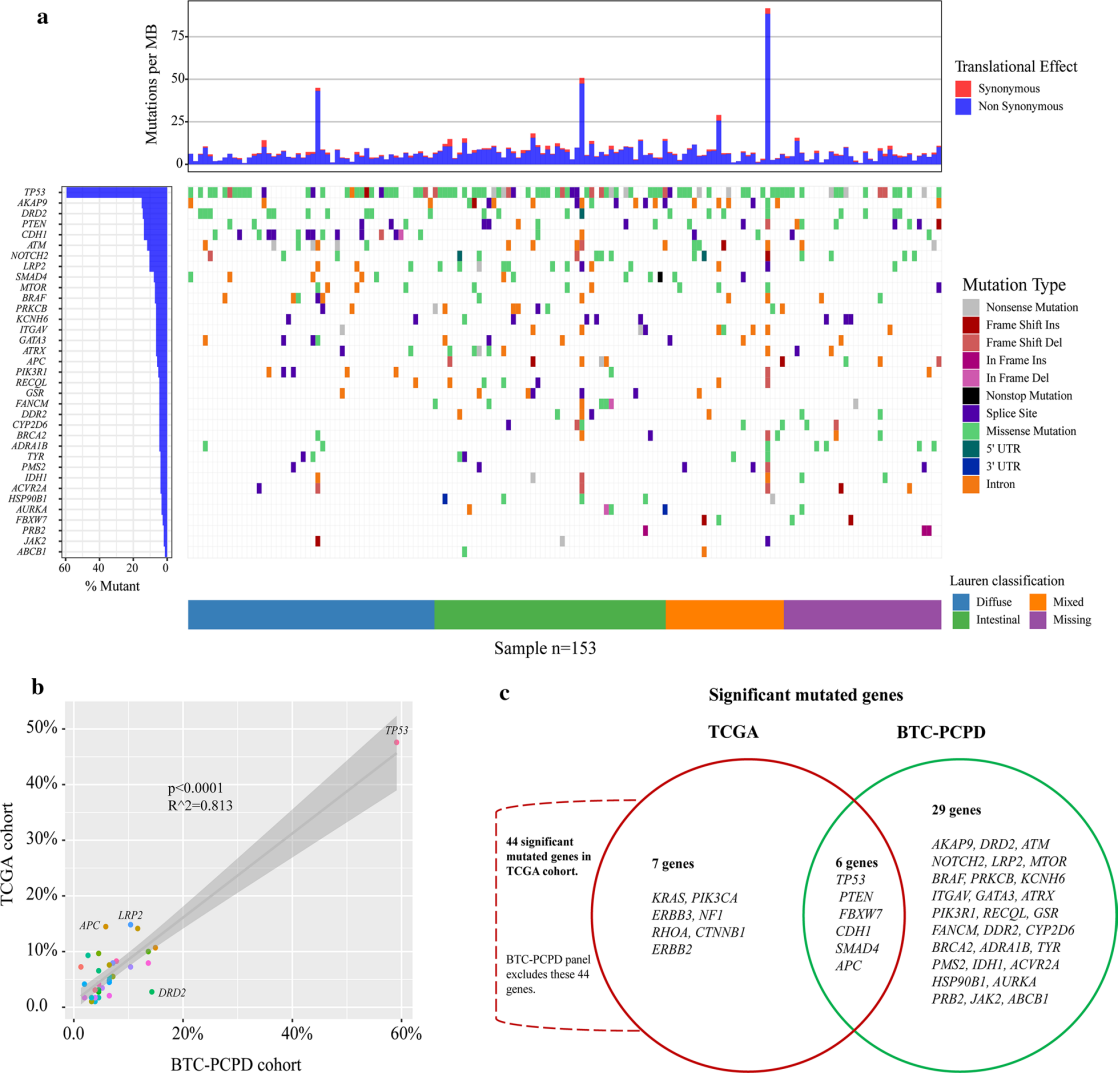
Significantly mutated genes of gastric cancer by BTC-PCPD. **a** Significantly mutated genes, identified by MutSigCV, are ranked by mutant frequency and Lauren classification. The bars in top represent tumor mutation burden (TMB, mutations per Mb). **b** Comparison of frequency of 35 significantly mutated genes identified by this study between TCGA cohort and BTC-PCPD cohort. **c** Venn diagram of significantly mutated genes identified by MutSigCV between TCGA cohort and BTC-PCPD cohort. TCGA, The Cancer Genome Atlas

We used MutSigCV tool to define significantly mutated genes in 153 gastric tumors, and identified 35 significantly mutated genes (Fig. 1a). The five most frequently mutated genes were *TP53* (59.09%), *DRD2* (14.29%), *CDH1* (13.64%), *AKAP9* (14.93%) and *ATM* (11.69%) (Fig. 1a). Lollipop plots showed the type and location of all significant gene mutations (Additional file 2). Obviously, the BTC-PCPD panel results were highly consistent with the TCGA finding, exhibiting strong concordance of the 35 mutated genes and population frequencies of mutations detected (Fig. 1b), but relatively weaker concordance of all somatic mutated genes (Additional file 3). But there were still some deflected genes such as *APC*, *LRP2*, *DRD2* (Fig. 1b). *TP53* was the most frequently mutated gene in TCGA (47.59%) [4] and BTC-PCPD cohort (59.09%). We found *TP53* tended to mutate in male (p = 0.025) gastric cancer patients and patients whose tumor located in cardia (p = 0.011) (Table 1).

Comparative analysis of significantly mutated genes was performed between BTC-PCPD cohort and TCGA cohort of gastric cancer. We observed important differences between two cohorts. 13 significantly mutated genes reported by TCGA were covered by BTC-PCPD panel. Among them, only 6 genes were analyzed as significantly mutated genes by MutSigCV in BTC-PCPD cohort, including *TP53* (59.09%), *PTEN* (13.64%), *FBXW7* (2.60%), *CDH1* (13.64%), *SMAD4* (7.79%) and *APC* (5.84%) (Fig. 1c). Compared with TCGA result, 29 novel significantly mutated genes were identified in this study (Fig. 1c), and the top five most frequently mutated genes were *AKAP9* (14.94%), *DRD2* (14.29%), *ATM* (11.69%), *NOTCH2* (10.39%) *and LRP2* (10.39%).

**Fig. 2:**
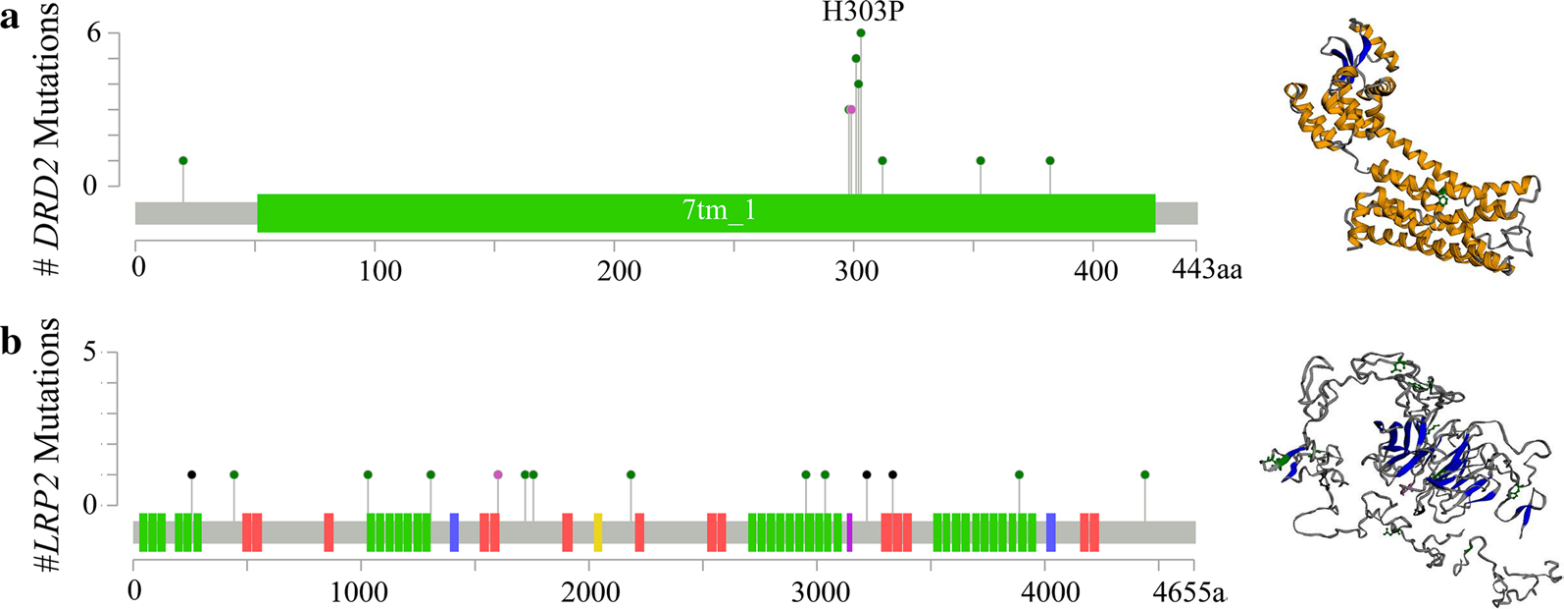
The proportion of mutations and protein structure of **a** *DRD2* and *LRP2* (**b**)

We first focused on *DRD2* among the 29 novel significantly mutated genes. *DRD2* gene is located in 11q23.2, encoding D2 subtype of the dopamine receptor. *DRD2* was found to mutate at a higher frequency in this study (14.29%) than in TCGA cohort (2.76%) (Fig. 1b). Almost all mutation sites were located in 7 transmembrane receptor (7tm_1) functional region with dense distribution, and *DRD2* p.H303P was the mutational hotspot (Fig. 2a). Dopamine (DA), a neurotransmitter, has been reported to play an important role in tumor progression [13]. Previous studies indicated prominence of DR signaling in human cancer and cancer progression. H Huang’s study indicated dopamine D2 receptor suppresses invasion and migration of gastric cancer cells via inhibition of EGFR/AKT/MMP-13 pathway [14]. While, Mu’s study reported that high expression of*DRD2* is correlated with poor prognosis of gastric cancer [15]. Dopamine D2 receptor was also reported to serve as biomarker of cancer stem cells for diverse malignancies. Dopamine D2-like receptor (D2DR) antagonists such as thioridazine could selectively target cancer stem cell, while having no effect on normal blood stem cells [16].

*LRP2* is located in 2q31.1, encoding low density lipoprotein-related protein 2 (megalin), which is critical for the reuptake of numerous ligands, including lipoproteins, sterols, vitamin-binding proteins, and hormones [17]. Unlike the case in *DRD2*, mutation sites were scattered in *LRP2* (Fig. 2b). *LRP2* is involved in cell uptake of vitamin D [18]. Megalin is acell surface protein abundantly present in the kidney that binds DBP (vitamin D binding protein) to mediate internalization of 25(OH)D into the cytosol [17]. *LRP2* variants have previously been associated with prostate cancer [19] and pancreatic cancer risk [20]. Ge et al. first reported *LRP2* as a significantly mutated gene (7.7%) in gastric cancer in 2017 [7]. But it is controversial whether vitamin D intake, serum vitamin D levels and vitamin D pathway were associated with gastric cancer [21, 22].

### Somatic copy number alterations (SCNAs) detection

**Fig. 3:**
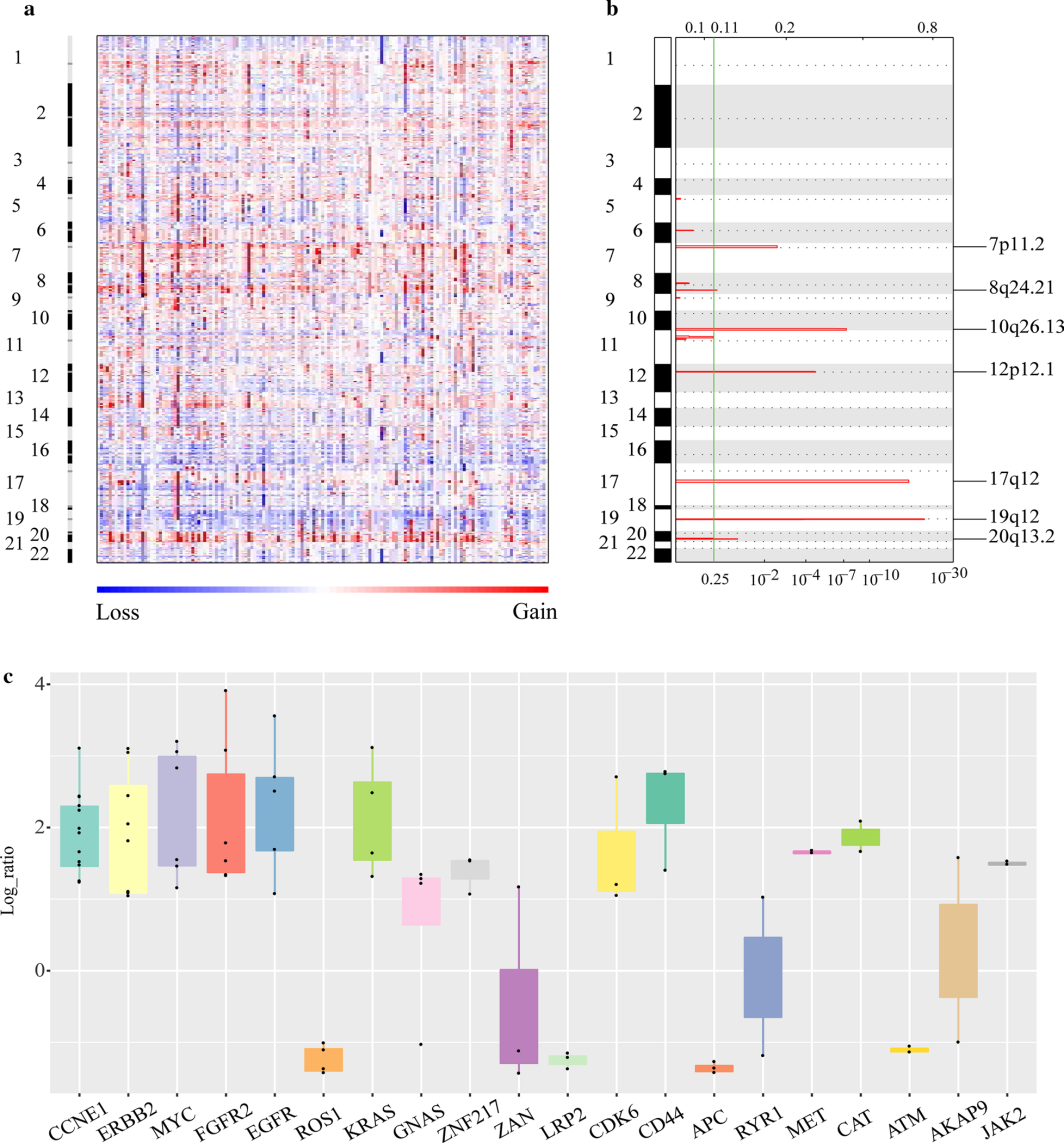
Somatic copy number alterations (SCNAs). **a** SCNAs in tumors are plotted by chromosomal location (vertical axis) by GISTIC 2.0 analysis of the entire dataset. **b** Focal amplifications. Chromosomal locations of significantly recurring focal amplifications by false discovery rates using GISTIC 2.0. Annotated peaks have an FDR < 0.25. No significantly recurring focal deletions were annotated. **c** The top 20 frequency genes

153 tumors were profiled for SCNAs of 22 autosomes (Fig. 3a). There were several significant peaks of copy number gain (Fig. 3b) including 7p11.2 (*EGFR*), 8q24.21 (*MYC*), 10q26.13 (*FGFR2*, *MMP21*), 12p12.1 (*KRAS*), 17q12 (*ERBB2*, *CDK12*, *GRB7*), 19q12 (*CCNE1*), and 20q13.2 (*ZNF217*). Figure 3c showed the top twenty SCNA genes according to frequency in samples. Among them, amplifications were detected in 13 genes, while deletions were found in 5 genes. Both amplification and deletion were identified in*RYR1* and *AKAP9* genes. Amplifications mainly targeted well-known oncogenes such as *EGFR*, *ERBB2*, *KRAS*, *MYC*, *CCNE1*, *JAK2*, *FGFR2* et al. 12 gastric tumor tissues harbored *CCNE1* amplifications (7.84%), which was the most frequent genes with SCNA in this study, and with similar frequency (10.58%) in TCGA cohort [4]. Amplification of *CCNE1* is associated with poor outcome in gastric [23], breast [24], and ovarian cancer [25, 26]. *ERBB2* amplification was detected in 8 gastric tumor tissues (5.23%). Among 8 samples with *ERBB2* amplifications, 6 samples (75%) were positive by immunohistochemical (IHC) of erb-b2 receptor tyrosine kinase 2 (Additional file 4), which was the target of trastuzumab. Additionally, we also found amplification of *CD44*, which is a gastric stem cell marker [27].

We identified deletions in several genes including *ROS1*, *ZAN*, *APC*, *LRP2*, *ATM* et al. (Fig. 3c). *ROS1* encodes an orphan receptor tyrosine kinase related to anaplastic lymphoma kinase (ALK) [28]. It is well-known that *ROS1* could be activated by chromosomal rearrangement in various human cancers such as non-small-cell lung cancer (NSCLC) [29]. *ROS1* deletion was found in 4 samples in this study, while *ROS1* amplification and deletion were reported in 2 samples each in TCGA cohort of 293 samples [4]. *ROS1* amplifications and deletions were also reported to be heterogeneous in NSCLC, which had no impact on overall survival [10]. In this study, both *LRP2* and *APC* deletion were detected in three samples, and *ATM* deletion was found in 2 samples, which were also reported deletions by TCGA.

### Enrichment of somatic mutated genes by GO and KEGG analysis

**Fig. 4:**
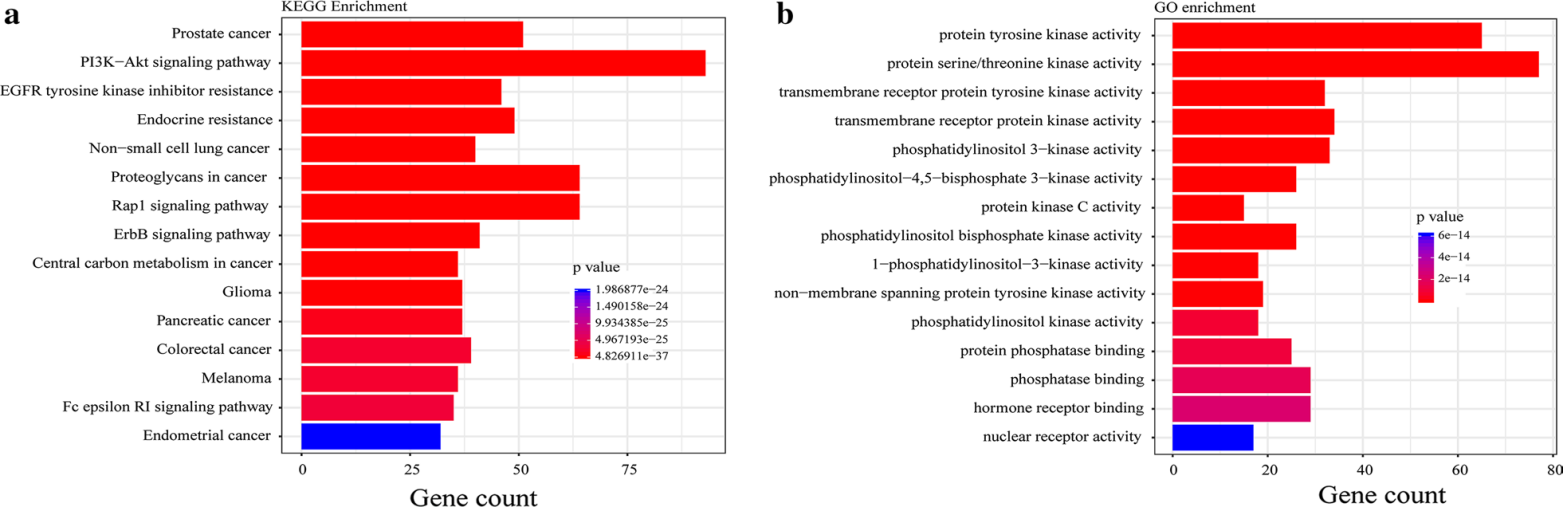
Signaling pathways by **a** KEGG and functional terms by **b** GO enrichment of somatic mutated genes. Gene count: the number of mutated genes enriched in this pathway or functional term

To better understand the biological function of mutated genes, GO and KEGG enrichment analysis were performed. Altered signaling pathways included cell movement related pathways (adherens junction, focal adhesion and regulation of actin cytoskeleton), PI3K–Akt signaling, ErbB signaling, VEGF signaling, MAPK signaling, Ras signaling, p53 signaling, JAK-STAT pathway, and other well-known pathways. Figure 4 showed the top 15 pathways according to gene count and p value enriched by KEGG (Fig. 4a) and GO (Fig. 4b). In particular, we focused on alteration in PI3K–Akt signaling pathway, which is a key driver in carcinogenesis. As previous studies reported, PI3K signaling pathway was recurrently activated in gastric cancer [4], colorectal cancer [30], breast cancer [31] and other cancers [32, 33]. 93 genes with somatic mutations were enriched to PI3K–Akt signaling pathway. 13.07% patients harbored mutations in *PTEN* gene, which is a tumor suppressor of the PI3K–Akt pathway with eventual functional inactivation of this gene product. Involvement of several molecules of the PI3K–Akt pathways in GC carcinogenesis has eventually led to development of both single, as well as recently, dual inhibitors essential for molecular targeted therapy for GC, including pan-class I inhibitors, isoform specific PI3K inhibitors, Akt inhibitors, dual Akt/mTOR inhibitors [34]. GO enrichment result showed that most of functional categories were related with kinase activity (Fig. 4b), such as protein tyrosine kinase activity.

### Clinical actionability for targeted therapy

**Fig. 5:**
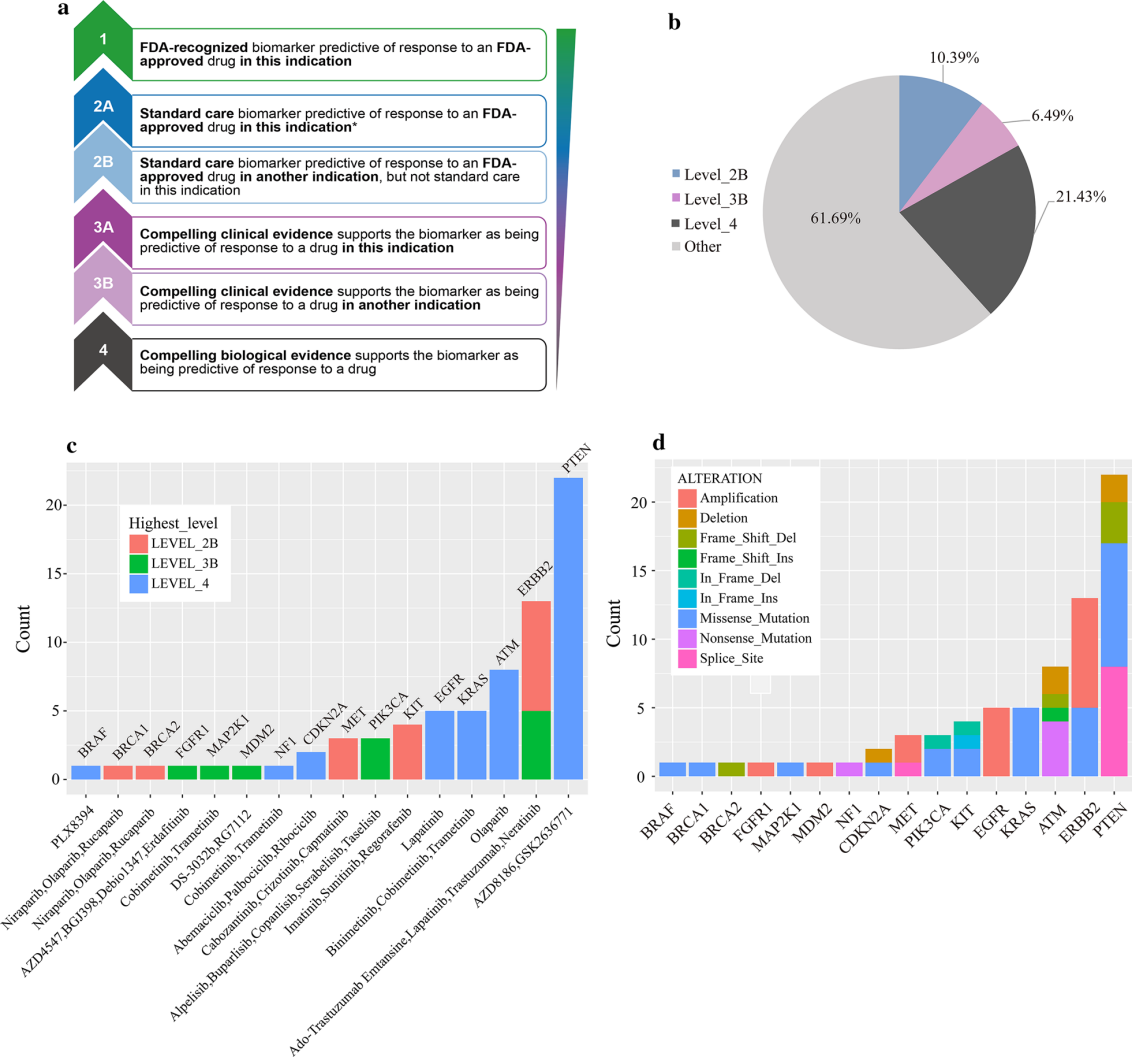
Clinical actionability of somatic alterations revealed by BTC-PCPD. **a** Alterations were defined based on their clinical evidence according to OncoKB. **b** Samples were assigned to the highest level of actionable alterations. **c** Distribution of levels of actionable alterations. **d** Distribution of alteration types

To evaluate the clinical utility of prospective molecular profiling to guide treatment decisions, we used OncoKB (http://oncokb.org/) to group all mutations into various levels according to evidence of clinical actionability (Fig. 5a). Altogether, 38.31% of patients harbored at least one actionable alteration (Fig. 5b). We found a group of gene mutations as standard care biomarkers for an FDA-approved drug in another indication. 10.39% tumors harbored level_2B gene alterations (Fig. 5b) including In-frame shift of *KIT*, frameshift of *BRCA2*, SNVs of *KIT*, *BRCA1* and *MET*, and amplifications of *ERBB2* and *MET* (Fig. 5c, d). Level_3B accounted for 6.49% (Fig. 5b), including missense mutations of*ERBB2*, *PIK3CA*, *MAP2K1*, In-frameshift of PIK3CA, and amplifications of*FGFR1* and *MDM2* (Fig. 5c, d). Level_4 accounted for 21.43% (Fig. 5b), including SNVs in *PTEN*, *BRAF*, *KRAS*, *ATM*, *CDKN2A*, amplification of *EGFR* and deletions of *PTEN* (Fig. 5c, d). Specifically, we found 22 actionable alterations of *PTEN* in 10 patients, which was the target of PI3K inhibitors AZD8186, as well as GSK2636771, and was classified as Level_4 (Fig. 5c).

#### Tumor mutation burden of gastric cancer

**Fig. 6:**
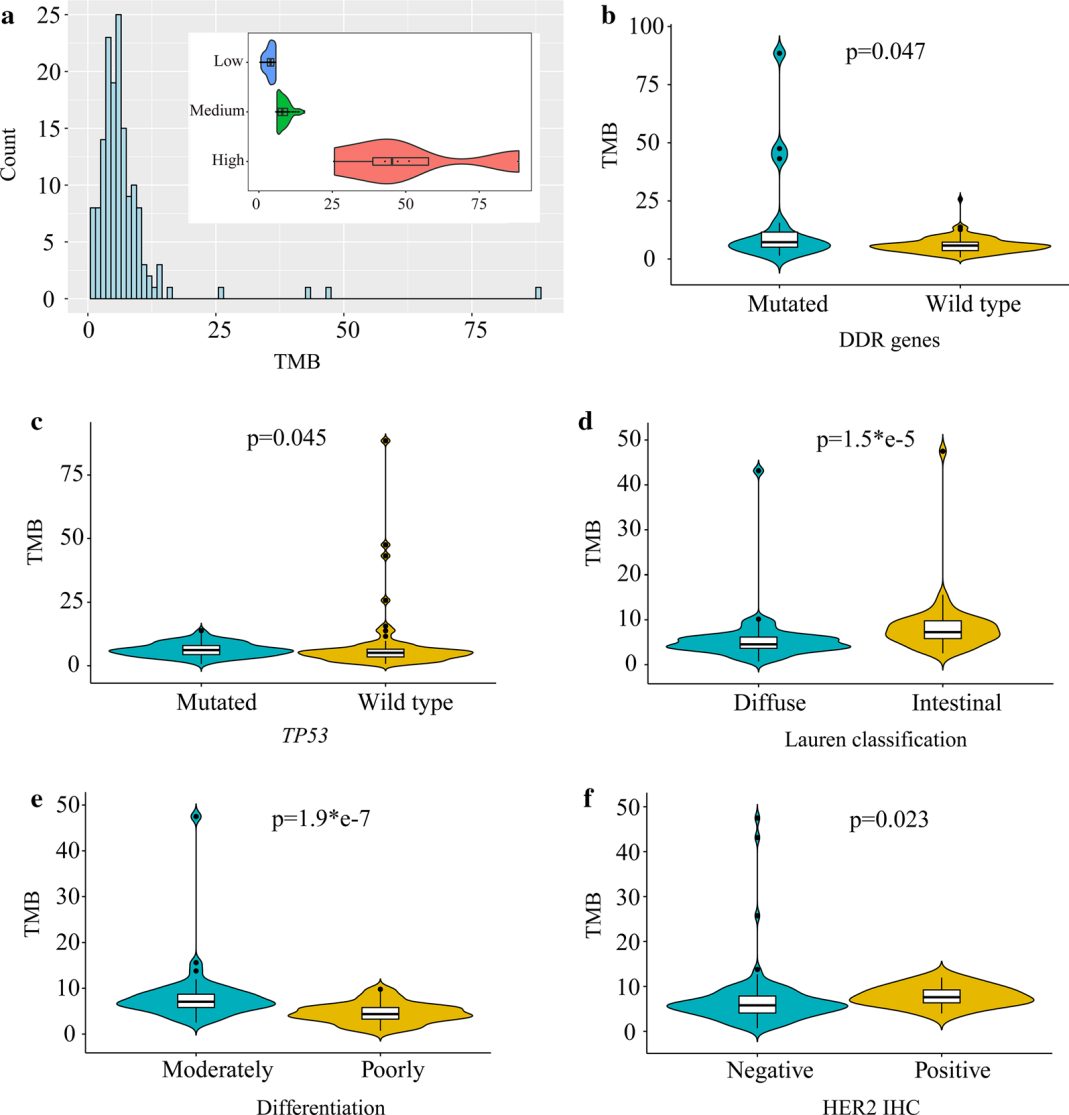
Tumor mutation burden of gastric cancer. **a** The distribution of TMB across all tumors, using a threshold of low (1–5 mutations/Mb), intermediate (6–19 mutations/Mb), and high (≥ 20 mutations/Mb). Non-parametric test of TMB according to DDR genes (**b**) and *TP53* (**c**) genotype, Lauren classification (**d**), differentiation (**e**), and HER2 status (**f**)

High tumor mutation burden (TMB) is an emerging biomarker of sensitivity to immune checkpoint inhibitors such as PD-1 and PD-L1 blockade immunotherapy. NGS panel is a reliable technology to analyze TMB, which was well reported by previous studies [35, 36]. In this study, we evaluated TMB of all 153 gastric tumors by BTC-PCPD panel (612 genes, 2.75 Mb capture region). The median TMB of all samples was 5.801 (range 0.725–88.470) mutations/Mb (Fig. 6a). To the best of our knowledge, there is no uniform standard for high TMB yet. Currently, TMB levels could be divided into three groups based on the Foundation Medicine official reports: low (1–5 mutations/Mb), intermediate (6–19 mutations/Mb), and high (≥ 20 mutations/Mb) [37], which was also applied in this study. We thus divided 4 patients (2.61%) to high TMB, 63 patients (41.18%) to intermediate TMB, 86 patients (56.21%) to low TMB (Fig. 6a). Zehir’s study using MSK-IMPACT panel indicated that the threshold for tumors with a high TMB was 13.8 mutations/Mb [6]. Accordingly, 5 tumors with TMB above 13.8 mutations/Mb was identified as high TMB in this study.

Mutations in DNA damage repair (DDR) genes occur as both germline polymorphisms and somatic mutations, including mismatch repair (MMR), base excision repair (BER) and homology-dependent recombination (HR), nucleotide excision repair (NER) and so on [38]. Defects in DNA replication, if not properly repaired, can lead to increased somatic mutation rate [35]. Twenty-seven genes within BTC-PCPD panel were previously identified as DDR related [38]. In this study, we analyzed deleterious germline mutations (potential pathogenic germline variants) and somatic mutations in DDR genes. Tumors with somatic mutations in DDR genes got higher TMB than wild-type tumors (p-value = 0.047) (Fig. 6b). But we found no difference on TMB of DDR genes with germline mutations.

There were significant associations between TMB and *TP53* genotype (p = 0.045, Fig. 6c), Lauren classification (p = 1.5e−5, Fig. 6d), differentiation (p = 1.9e−7, Fig. 6e), as well as HER2 IHC status (p = 0.023, Fig. 6f). *TP53*-mutated tumors had higher median TMB, but lower mean TMB than *TP53* wild-type tumors, and the similar phenomenon was found in TCGA dataset. In this study, all 5 high-TMB tumors (> 13.8 mutations/Mb) gathered in *TP53* wild-type tumors. In TCGA dataset, we also found more high-TMB tumors with *TP53* wild-type, but the difference was not significant (p = 0.088) (Additional file 5). Intestinal type and moderately differentiated tumors got higher TMB than diffuse and poorly differentiated type. HER2 positive tumors had higher median TMB value, but all high-TMB tumors belonged to HER2 negative tumors.

## Discussion

Gastric cancer is a genomically heterogeneous disease. Here, we characterized and provided extensive data describing tumor genomic mutation profile across 153 gastric cancer patients in the clinical setting. We assessed SNVs, InDels, SCNAs and TMB of tumors by BTC-PCPD panel, a target capture assay, which contributed to guiding the selection of genomically matched therapies for patients. Altogether, 38.31% of patients harbored at least one actionable alteration (Fig. 5) for treatment according to clinical evidence, which were classified into Level_2B, Level_3B, and Level_4. Unfortunately, no gene mutation was classified as Level_1 (FDA-recognized biomarker for an FDA-approved drug in the same indication). Although few patients with actionable alterations received genomically matched therapy because of medical, logistical and economic considerations, a group of patients still had choice to participate in clinical trials of matched targeted drugs, or try drugs in other indications.

We observed a wide range of TMB (0.725–88.470 mutations/Mb), and five patients were identified as high TMB (> 13.8 mutations/Mb), with which patients may respond to immune-checkpoint inhibitors. It is hypothesized that highly mutated tumors are more likely to harbor neoantigens, which make them targets of activated immune cells. Increased mutation rate is well-characterized feature of human cancer. We found high TMB tumors tended to be enriched in tumors with wild-type *TP53*, somatic mutated DDR genes, and negative HER2. Previous studies reported that advanced urothelial carcinoma patients with DDR genes alterations could benefit from Platinum-based [39] and PD-1/PD-L1 blockade [40] therapy.

In this study, we identified 35 significantly mutated genes, as expected, the tumor suppressor gene *TP53* is the top frequently mutated gene. We found that *TP53* genotype was not only associated with clinical characteristic (gender and tumor location) but also tumor mutation burden (TMB). *TP53* mutated tumors had higher median TMB than wild-type tumors. But interestingly, particular high TMB (TMB > 13.8 mutations per Mb) was enriched in *TP53* wild type tumors. Loss of function mutations in *TP53* are very common in various cancers and are a somatic marker of elevated mutation rate [41]. Instead, in lung adenocarcinoma, previous studies reported *TP53* mutated more frequently in high TMB tumors (define: > 4.85 mutations/Mb) [42], and had potential predictive value for response to PD-1 blockade immunotherapy. In addition, we found 29 novel significantly mutated genes which were not reported as significantly mutated genes in TCGA study.

## Conclusions

Taken together, we developed a hybridization capture-based NGS panel (BTC-PCPD), drew a comprehensive mutational landscape of 153 gastric tumors, and demonstrated utility of massively parallel DNA sequencing of tumors to guide clinical management. There are limitations in our study. Survival analysis was not performed because of short time from patient enrollment. In the future, we will perform long term follow-up for these GC patients and recruit more new GC patients.

## Additional files

**Additional file 1.** Gene list of BTC-PCPD panel.

**Additional file 2.** The proportion of mutations of 35 significantly mutated genes.

**Additional file 3.** Comparison of frequency of all mutated genes identified by this study between TCGA cohort and BTC-PCPD cohort.

**Additional file 4.** Immunostaining for the ERBB2 protein, IHC score 3+.

**Additional file 5.** Comparison of TMB level between *TP53* mutated andwild type tumors in gastric cancer of TCGA dataset.

## Data Availability

Data sharing is applicable to this article.

## Abbreviations

GC: gastric cancer
NGS: next-generation sequencing
WES: whole-exome sequencing
BTC-PCPD: Biotecan PanCancer Panoramic Detection
FFPE: Formalin-Fixed and Paraffin-Embedded
TMB: tumor mutation burden
CNA: copy number variation
GO: gene ontology
KEGG: Kyoto Encyclopedia of Genes and Genomes
MSI: microsatellite instability
TCGA: The Cancer Genome Atlas
