# Supplementary figures and images for: Mutational landscape of gastric cancer and clinical application of genomic profiling based on target next-generation sequencing
Source: J Transl Med. 2019 Jun 4;17:189. doi: 10.1186/s12967-019-1941-0 (PMC6549266; doi:10.1186/s12967-019-1941-0)

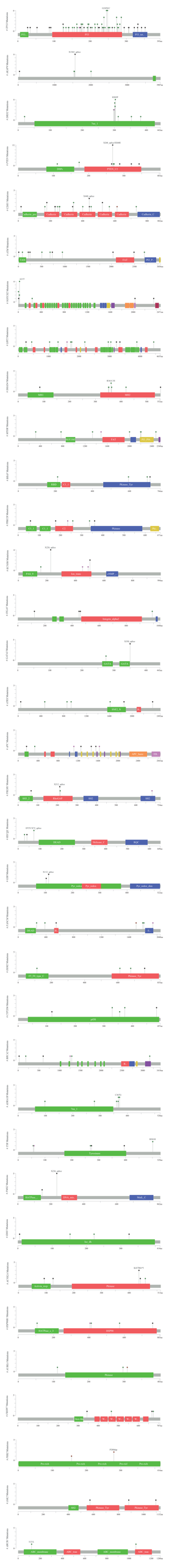

Supplement: Supplementary file 2 — Additional file 2. The proportion of mutations of 35 significantly mutated genes. [file 12967_2019_1941_MOESM2_ESM.pdf]

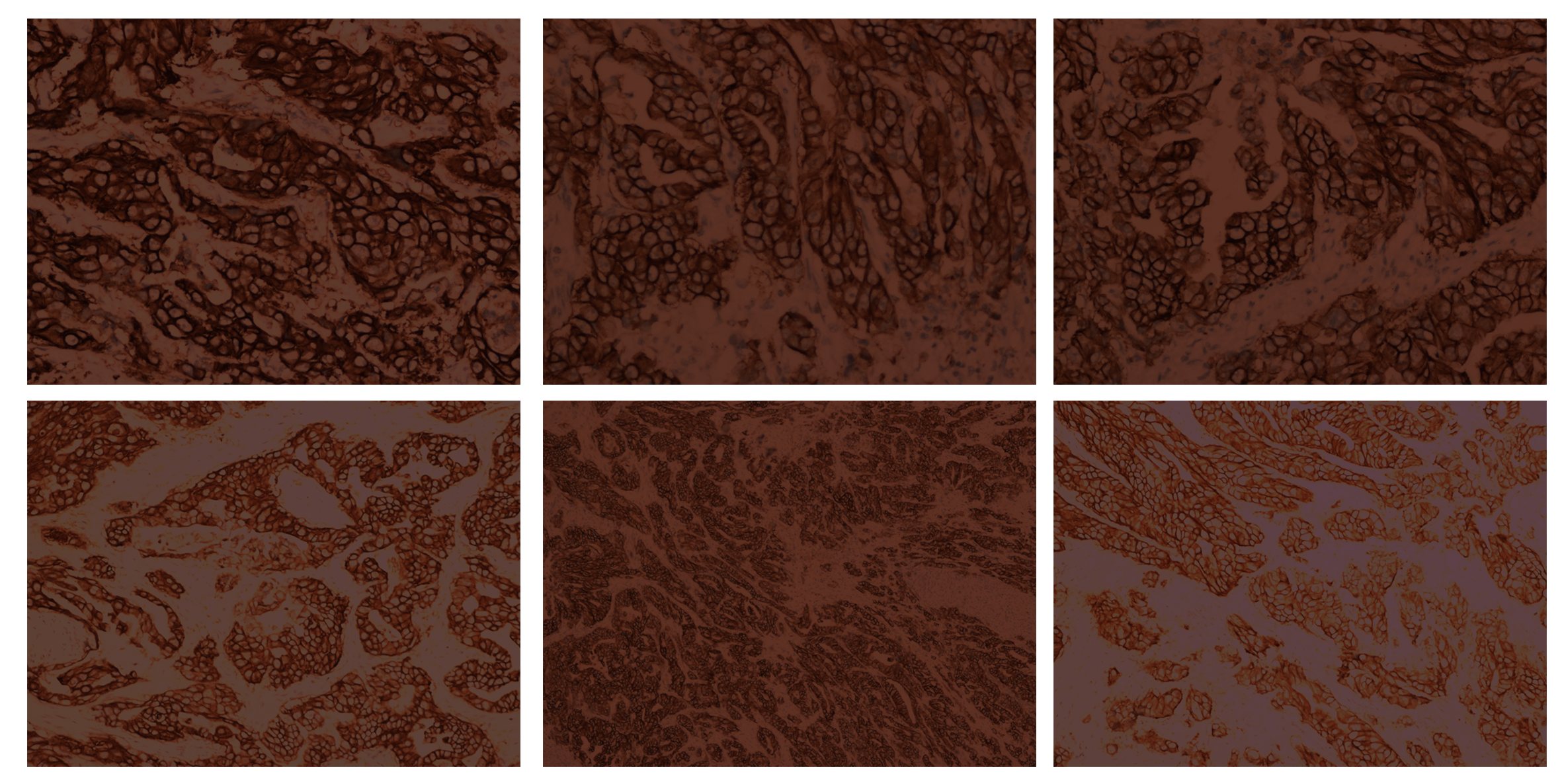

Supplement: Supplementary file 4 — Additional file 4. Immunostaining for the ERBB2 protein, IHC score 3+. [file 12967_2019_1941_MOESM4_ESM.tif]

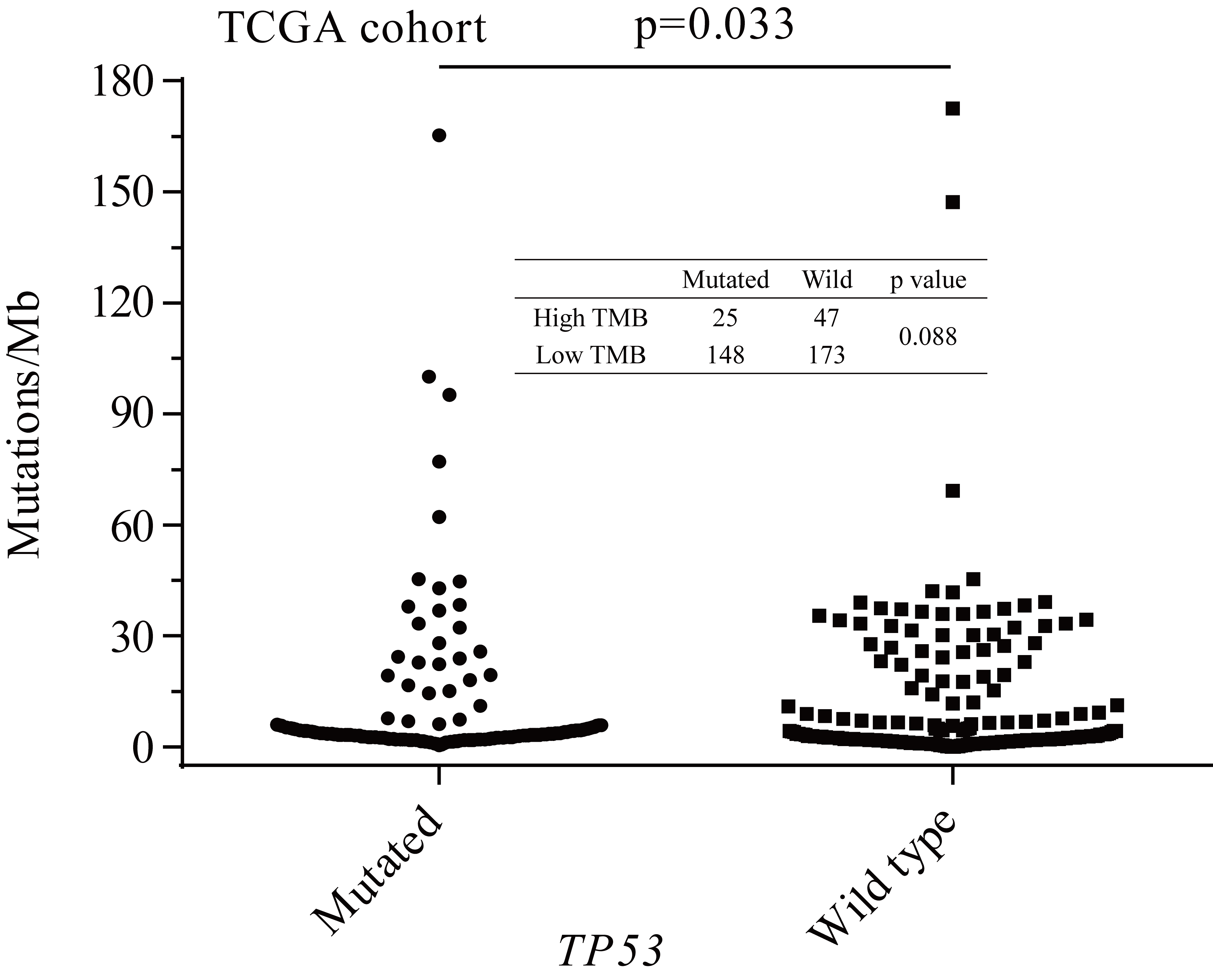

Supplement: Supplementary file 5 — Additional file 5. Comparison of TMB level between TP53 mutated andwild type tumors in gastric cancer of TCGA dataset. [file 12967_2019_1941_MOESM5_ESM.tif]
